# Docking of LDCVs Is Modulated by Lower Intracellular [Ca^2+^] than Priming

**DOI:** 10.1371/journal.pone.0036416

**Published:** 2012-05-10

**Authors:** Mathias Pasche, Ulf Matti, Detlef Hof, Jens Rettig, Ute Becherer

**Affiliations:** Physiologisches Institut, Universität des Saarlandes, Homburg/Saar, Germany; University of Waterloo, Canada

## Abstract

Many regulatory steps precede final membrane fusion in neuroendocrine cells. Some parts of this preparatory cascade, including fusion and priming, are dependent on the intracellular Ca^2+^ concentration ([Ca^2+^]_i_). However, the functional implications of [Ca^2+^]_i_ in the regulation of docking remain elusive and controversial due to an inability to determine the modulatory effect of [Ca^2+^]_i_. Using a combination of TIRF-microscopy and electrophysiology we followed the movement of large dense core vesicles (LDCVs) close to the plasma membrane, simultaneously measuring membrane capacitance and [Ca^2+^]_i_. We found that a free [Ca^2+^]_i_ of 700 nM maximized the immediately releasable pool and minimized the lateral mobility of vesicles, which is consistent with a maximal increase of the pool size of primed LDCVs. The parameters that reflect docking, i.e. axial mobility and the fraction of LDCVs residing at the plasma membrane for less than 5 seconds, were strongly decreased at a free [Ca^2+^]_i_ of 500 nM. These results provide the first evidence that docking and priming occur at different free intracellular Ca^2+^ concentrations, with docking efficiency being the most robust at 500 nM.

## Introduction

Before fusion with the plasma membrane (PM), secretory vesicles have to complete several steps, namely docking and priming. The least understood stage is docking due to a lack of suitable methods for measuring this activity [1]. Until now, docked vesicles were typically identified in electron micrographs by their proximity to the PM. The criteria for such “morphologically” docked vesicles vary, with distances as far as 30 nm from the PM and as close as visibly touching the PM [1], [2]. Additionally, electron micrographs do not allow the differentiation of docked and primed vesicles [3]. Hence little is known about the functional characteristics of docked vesicles. Because total internal reflection fluorescence (TIRF) microscopy allows the observation of large dense core vesicles (LDCVs) that reside at the PM, it is ideally suited for observing both docking and priming [4], [5], [6].

In contrast to the static snapshots obtained with electron microscopy, TIRF-microscopy gives real-time access to dynamic processes that occur close to the PM. For example, LDCVs of neuroendocrine cells, such as chromaffin cells, can be observed in vivo before secretion. Their residence time and their mobility at the PM allow differentiation between docked and primed LDCVs [7], [8], [9]. Thus, the functional characteristics of docked vesicles can now be determined to study how docking occurs.

Intracellular Ca^2+^ plays a role in several stages of exocytosis, triggering fusion at micromolar concentrations and promoting priming at nanomolar concentrations [10], [11], [12], [13]. However, the role of Ca^2+^ in docking remains largely unknown. We assessed the effect of varying intracellular Ca^2+^ concentrations ([Ca^2+^]_i_) on both docking and priming in bovine chromaffin cells using TIRF-microscopy and simul­taneous patch-clamp recordings [14]. Patch-clamp recording in whole cell configuration allowed us to monitor secretion and maintain [Ca^2+^]_i_ at the desired level, whereas TIRF-microscopy enabled the observation of fluorescently labeled LDCVs in the vicinity of the PM.

Changing [Ca^2+^]_i_ from 100 nM to 700 nM resulted in an increased size of the immediately releasable pool (IRP) of vesicles and decreased lateral mobility of LDCVs, indicating enhanced priming. Maximal axial immobilization of LDCVs was observed when the [Ca^2+^]_i_ was maintained at 500 nM. Additionally, at the same [Ca^2+^]_i_, the number of LDCVs retained for an intermediate time at the PM was reduced to a minimum. Both of these results indicate that increasing the [Ca^2+^]_i_ to 500 nM induced optimal docking. These findings show for the first time that Ca^2+^ modulates docking and that this modulation occurs at a different [Ca^2+^]_i_ than priming.

## Results

### Increased Concentration of Free Intracellular Ca^2+^ Led to Increased Releasable Pool Size

**Figure 1:**
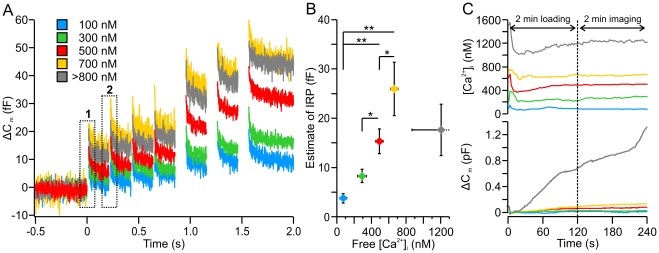
Increased IRP at intermediate [Ca^2+^]_i_. (**A**) Averaged traces of membrane capacitance recordings. Cells were stimulated by four 10-ms and three 100-ms depolarizations from resting potential (-70 mV) to 0 mV at 100-ms intervals. The different [Ca^2+^]_i_ are color coded (see inset). (**B**) Based on the responses to the first pair of short stimuli (A, boxes 1 and 2), the maximal size of the IRP was calculated and plotted versus the average [Ca^2+^]_i_. The IRP could not be reliably determined for [Ca^2+^]_i_>800 nM. The number given in B represents a close approximation. Error bars for IRP indicate s.e.m., whereas error bars for [Ca^2+^]_i_ indicate s.d. to better display the range of free [Ca^2+^]_i_ within a bin. (**C**) Constant secretion of LDCVs during imaging period was elicited only if the [Ca^2+^]_i_ was maintained above 800 nM. Membrane capacitance recordings (bottom panel) and traces of the corresponding [Ca^2+^]_i_ (top panel) during the loading and TIRF imaging phase of the experiment. N_100nM_: 23; N_300nM_: 13; N_500nM_: 26; N_700nM_: 14; N_>800 nM_: 16; *P<0.05; **P<0.01.

LDCVs in bovine chromaffin cells were stained by over-expressing neuropeptide Y (NPY) fused to the red fluorescent protein mCherry, and their mobility near the PM was visualized by TIRF-microscopy. In order to control the intracellular medium we performed patch-clamp measurements in whole cell configuration and used different pipette solutions containing various amounts of free Ca^2+^. The [Ca^2+^]_i_ was measured with the ratiometric Ca^2+^ indicators Fura2 and FuraFF. We reduced Ca^2+^ influx with an extracellular medium containing 1 mM Ca^2+^ ([Ca^2+^]_e_) and 4 mM Mg^2+^ ([Mg^2+^]_e_). This approach allowed us to set a constant [Ca^2+^]_i_ ranging from resting levels of approximately 100 nM up to the low micromolar range at which secretion is initiated. To examine the physiological significance of the various calcium concentrations, we loaded the cell for 2 min to establish constant [Ca^2+^]_i_ and then imaged the fluorescently labeled LDCVs for additional 2 min. [Ca^2+^]_i_ and membrane capacitance were measured throughout the experiment (Figure 1C). Finally, a train of depolarizations was applied to induce exocytosis and the membrane capacitance was recorded with high time resolution. Since all measurements (TIRF-microscopy, membrane capacitance, [Ca^2+^]_i_) were performed simultaneously, the identical sets of cells were used to generate all the parameters analyzed in this work.

As shown in Figure 1A, the exocytotic response increases with increasing pre-stimulus [Ca^2+^]_i_. We pooled cells with similar [Ca^2+^]_i_ and sorted them into five bins in which the average [Ca^2+^]_i_ (±s.d.) of the cells was 73±31, 293±49, 492±39, 665±62, and 1206±336 nM (Figure 1B). For simplification, we named these bins 100, 300, 500, 700, and >800 nM. To estimate the Ca^2+^-dependence of priming, the size of the rapidly releasable pool (RRP) of vesicles is usually determined. However, in order to tightly control the [Ca^2+^]_i_, we reduced the [Ca^2+^]_e_ and increased the [Mg^2+^]_e_, which substantially reduced the influx of Ca^2+^ and size of the Ca^2+^-microdomain during depolarization. Consequently, the apparent size of the RRP was also strongly diminished (data not shown). In contrast, vesicles in close proximity to the Ca^2+^ channels, such as vesicles belonging to the IRP, were less affected by the reduced size of the Ca^2+^-microdomain. Compared to the apparent size reduction of the RRP, the size of the IRP (Figure 1B) was consistent with previous measures of priming [15]. Using Eq. 1 in Material and Methods, we found that the size of the IRP increased from 3.8±1.0 fF at 100 nM [Ca^2+^]_i_ to 26.1±5.4 fF at 700 nM free [Ca^2+^]_i_ (N = 23 and 14, respectively, *P* = 0.007, Figure 1B). [Ca^2+^]_i_ >800 nM induced a slow but steady secretion (Figure 1C, bottom panel) that hindered proper determination of the IRP but appeared to lead to a reduction of its size. In summary, we found that increasing the [Ca^2+^]_i_ from 100 nM to 700 nM increased priming as previously reported [12].

### Lateral Mobility of LDCVs was Minimized at an [Ca^2+^]_i_ of 700 nM

**Figure 2:**
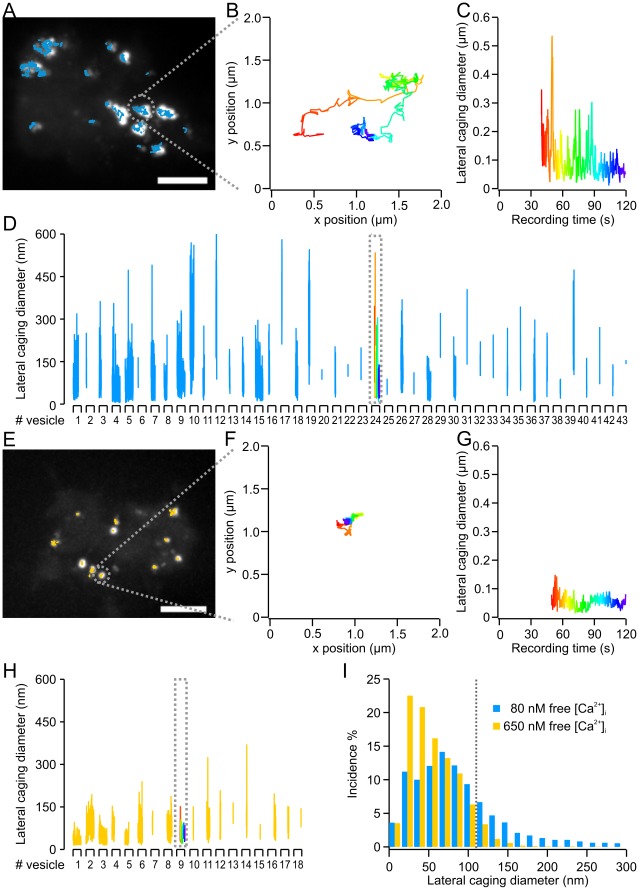
Increased free [Ca^2+^]_i_ led to a lateral immobilization of vesicles. (**A**) Overlay of the intensity projection of LDCV fluorescence with the trajectories (blue traces) obtained for the exemplary cell kept at an [Ca^2+^]_i_ of ≈80 nM. (**B**) Lateral movement of an exemplary LDCV chosen from the cell in (A) (grey dotted circle) and (**C**) the resulting CD vs. time. Time is color coded (red-yellow-green-blue). Note that this LDCV appeared in the evanescent wave only 40 s after starting the recording and remained until the end. (**D**) CD time course for each individual LDCV of the exemplary cell shown in (A). Each segment of the X-axis represents the 2 min recording time. The blue line above one segment corresponds to the CD versus time of one LDCV. The exemplary LDCV in (B, C) is displayed within the dotted grey box. Note that not all LDCVs remained in the evanescent field for the same amount of time. (**E**) Overlay of the intensity projection of LDCV fluorescence with the trajectories (orange traces) obtained for the exemplary cell kept at an [Ca^2+^]_i_ of ≈650 nM. (**F**) Lateral movements of an exemplary LDCV chosen from the cell in (E) (grey dotted circle) and (**G**) the resulting CD vs. time. Time is color coded (red-yellow-green-blue). Note that this LDCV appeared 49 s after starting the recording. (**H**) CD time course for each individual LDCV of the exemplary cell shown in (E). X-axis segments represent the 2 min recording time. The orange line above one segment corresponds to the CD versus time of one LDCV. The exemplary LDCV in (F, G) is displayed within the dotted grey box. (**I**) CD size distribution for the cells shown in (A) and (B). Bin size 16 nm. The histogram was normalized to the sum of all CDs measured per cell. The overall median value of both cells is 108 nm (gray dotted line). Note that the contribution of small CDs increased in the cell kept at ∼650 nM free Ca^2+^ compared to the cell maintained at low [Ca^2+^]_i_.

In an earlier study, we have shown that the mobility of LDCVs depends on their functional state [7]. In order to examine the calcium-dependence of LDCV mobility, we analyzed both lateral (parallel to the PM) and axial mobility (perpendicular to the PM). Lateral mobility was assessed by measuring the exact (X,Y) position of each LDCV over time. The resulting trajectory was examined by the caging diameter (CD) analysis [7], which assigned a CD value to each vesicle in every frame (Figure 2B, C, F, G). Due to the length of the time window, only LDCVs residing for more than 1 s were taken into account, excluding about one-third of all LDCVs in this analysis. However, these LDCVs were dim (Figure S1 and Table S1), indicating that they may not be stably docked and, therefore, irrelevant for analyzing priming or docking. In addition, their proportions remained constant at all [Ca^2+^]_i_.

**Figure 3:**
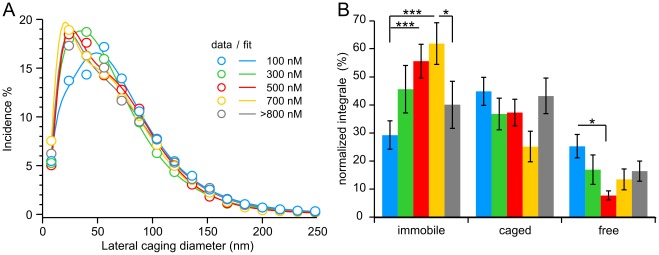
Reduced lateral mobility at a free [Ca^2+^]_i_ of 700 nM. (**A**) Average lateral CD size distribution for each [Ca^2+^]_i_. Open circles correspond to the average CD incidence in each bin (bin size 16 nm). Solid lines represent the best fit containing one log-normal (first) and two Gaussian components (second and third). Each component represents one mobility state: immobile, caged, or free. See Supplementary Figure S2 and Supplementary Table S2 for statistical analysis. (**B**) [Ca^2+^]_i_–dependence of the proportion of CDs in each mobility state. These values show the averages that were calculated from every component’s integral in the histogram of individual cells. N_100nM_/n: 23/719; N_300nM_/n: 13/403; N_500nM_/n: 26/695; N_700nM_/n: 14/309; N_>800 nM_/n: 16/390; *P<0.05; ***P<0.001.

In the two example cells in Figure 2, the cytoplasmic [Ca^2+^] of the first cell was 80 nM (Figure 2A; Movie S1) and 650 nM for the second cell (Figure 2E; Movie S2). The CD over time for each individual LDCV in these two cells is given in Figure 2D, H. Interestingly, the CDs of several LDCVs in the cell containing 80 nM free Ca^2+^ reached values above the overall median of 108 nm. In contrast, the CDs of LDCVs maintained at 650 nM [Ca^2+^]_i_ rarely exceeded this value (Figure 2I). The clear left shift of the CD histogram obtained at an [Ca^2+^]_i_ of 650 nM compared to 80 nM indicates a reduced LDCV mobility (Figure 2I). We then applied this analysis to each of the 13–26 cells at each [Ca^2+^]_i_, and the resulting histograms are shown in Figure 3A with circles representing the averaged data points (see Figure S2 and Table S2 for full statistical analysis). Due to the experimental protocol, the cells used for this analysis were the same cells as those shown in Figure 1. The average data were best fitted by the sum of one log-normal and two Gaussian distributions. We found that the components of the fit corresponded to the different mobility states previously shown to be related to undocked, docked and primed vesicles [7]. We termed these various mobility states free, caged, and immobile.

Through all [Ca^2+^]_i_, the log-normal component (immobile) had an average peak position at 30.5 nm, half-width of 0.97 and amplitude of 11.6%. The first Gaussian component (caged) had a peak position at 60.5 nm, half-width of 68.6 nm, and amplitude of 7.4%. The second Gaussian component (free) had a peak position at 186.8 nm, half-width of 83.0 nm, and amplitude of 0.2%. A log-normal fit is not described properly by peak position, amplitude, and width [16] and these values cannot be used for statistical purposes. Thus, we tested whether the proportion of CDs reflecting immobility, caged, or free mobility was affected by the [Ca^2+^]_i_ calculating the integral of each fit component for every cell. We found that as [Ca^2+^]_i_ was increased from 100 nM to 700 nM, the proportion of CDs reflecting immobility linearly increased with its maximum at 700 nM (Figure 3B). Raising the [Ca^2+^]_i_ above 800 nM reverted this effect. In fact, the proportion of CDs corresponding to immobility was more than doubled at 700 nM of free Ca^2+^ compared to 100 nM of free Ca^2+^ (*P*<0.001; N = 23 and 14 cells analyzed for 100 and 700 nM, respectively). Though no significant difference in the proportion of CDs representing caged mobility was found among all [Ca^2+^]_i_, the proportion of CDs reflecting caged mobility was the smallest at 700 nM free Ca^2+^. Finally, free mobility represented, at best, a quarter of all CDs and was also Ca^2+^-dependent. Overall, we observed that the maximum shift towards immobility was reached at 700 nM. Since immobile vesicles have been shown to be primed [7], our results indicate that the pool size of primed LDCVs was maximal at 700 nM [Ca^2+^]_i_.

### Lateral Mobility of LDCVs was Diminished Prior to Secretion

**Figure 4:**
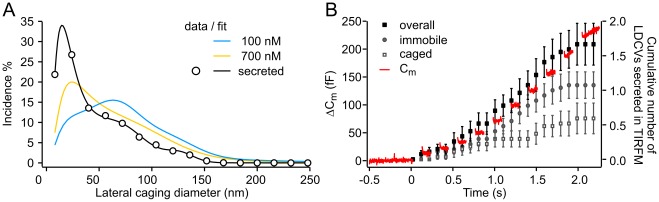
Secreted vesicles were immobile immediately before fusion. (**A**) Histogram of the averaged lateral CD distribution of secreted vesicles from cells at a pre-stimulus [Ca^2+^]_i_ of 590 nM (black). Data points are represented by circles, whereas solid lines represent the best fit by a single log-normal and two Gaussian components. Cells were stimulated by a train of ten depolarizations (5 Hz) 10 s before the end of the imaging period. The CD values were calculated from the last 15 s prior to fusion. The two fits obtained from cells kept at 100 nM (blue) and 700 nM (orange) [Ca^2+^]_i_ were plotted for comparison. (**B**) Average secretion of 19 cells stimulated 1 to 2 times. The increase in membrane capacitance is shown in red and the averaged cumulative number of exocytotic events visualized by TIRF-microscopy in black. The type of mobility of these LDCVs prior to secretion is indicated by grey filled circles (immobile) and grey empty boxes (caged). All curves were aligned to the onset of the stimulus. N_100nM_/n: 23/719; N_700nM_/n: 14/309; N_secreted_/n: 19/53.

We tested whether the fusion competence of a LDCV is related to its immobility under our current experimental conditions. In order to visualize exocytosis, we used cells transfected with NPY-mCherry via electroporation and maintained them for 3 to 4 days in culture to obtain a high density of stained LDCVs. To elicit strong secretion, we used an intracellular solution with high Ca^2+^ (Table 1, solution 3), an extracellular medium containing 2.5 mM [Ca^2+^]_e_ and 1.2 mM [Mg^2+^]_e_, and stimulated the cells with a train of 10 depolarizations (100 ms at 5 Hz). We selected cells (N = 19) in which the [Ca^2+^]_i_ was constant at 590±68 nM (±s.d.), i.e. where priming was induced. Finally, we back-tracked LDCVs for up to 15 s from the time of secretion and performed the CD analysis (Figure 4A).

**Table 1:** Composition of intracellular solutions (in mM).

	Solution number		
	1	2	3
Cs-glutamic-acid	135.2	132.8	130.6
Na_2_-GTP	0.3	0.3	0.3
HEPES	10	10	10
Fura2	0.4	0.4	0.4
Fura-FF	0.4	0.4	0.4
MgCl_2_	1	1	1
Mg-ATP	2	2	2
EGTA	2	3.8	5.9
Free EGTA	1.25	1.36	1.33
Ca^2+^	0.8	2.6	4.8
Free Ca^2+^	96•10^−6^	302•10^−6^	597•10^−6^

The CD histogram was fitted by the same sum of one log-normal and two Gaussian distributions as above (Eq. 8, Figure 3). The integrals of all three fit components were similar to the integrals of the fit components measured for resting cells maintained at 700 nM free Ca^2+^ (61, 26, and 12% for secreted vesicles, N_sec._ = 19 and 62±7, 25±5, and 13±4% for vesicles in resting cells, N_700nM_ = 14). Nonetheless, the log-normal component, which corresponds to immobility, left-shifted for secreted vesicles compared to vesicles in resting cells. We calculated a median CD value of the log-normal fit of 36.8±1.4 nm for resting cells, independent of the [Ca^2+^]_i_, compared to a median value of 21.6 nm for secreted vesicles. Thus, our data show that, within 15 s of their fusion with the PM, primed LDCVs were highly immobile.

In order to characterize the mobility state of LDCVs at the moment of fusion, we calculated the intersection points of each neighboring fit component of the secreted vesicles to outline borders in which the vesicle’s mobility state is defined (Figure 4A). The intersection points were 55.4 nm between the immobile and caged states (log-normal/first Gaussian) and 101.5 nm between the caged and free states (first/second Gaussian). Consequently, vesicles that were immobile or displaying caged mobility at the time of fusion had a CD value comprised between 0 and 55.4 nm or comprised between 55.4 and 101.5 nm, respectively. Using these data, we then analyzed the mobility of LDCVs during the last second before fusion. We found that two-thirds of the LDCVs were secreted from the immobile state, whereas the remaining third, except for one, were secreted from the caged mobility state (Figure 4B). All LDCVs secreted out of the RRP (i.e. within the first two depolarizations) were immobile. Taken together, the data show that LDCVs fuse with the PM from the primed immobile state when [Ca^2+^]_i_ is transiently elevated after a train of depolarizations.

### Axial Mobility of LDCVs was Minimized at [Ca^2+^]_i_ up to 500 nM

**Figure 5:**
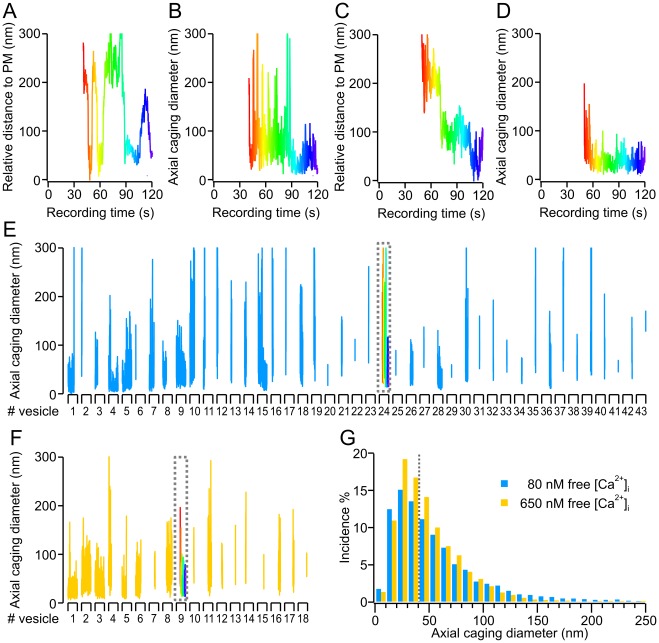
Increased free [Ca^2+^]_i_ led to axial immobilization of vesicles. (**A**) Relative axial movement over time of the exemplary LDCV chosen from the cell kept at a free [Ca^2+^]_i_ of ≈80 nM in Figure 2A (grey circle) and (**B**) the resulting CD vs. time. Time is color coded (red-yellow-green-blue) (**C**) Relative axial movement over time of the exemplary LDCV chosen from the cell kept at a free [Ca^2+^]_i_ of ≈650 nM in Figure 2E (grey circle) and (**D**) the resulting CD vs. time. Time is color coded (red-yellow-green-blue). (**E**) Relative axial CDs for each individual LDCV observed in the exemplary cell kept at a free [Ca^2+^]_i_ of ≈80 nM in Figure 2A and (**F**) the exemplary cell kept at a free [Ca^2+^]_i_ of ≈650 nM in Figure 2E. The results for each single LDCV were plotted against the recording time (2 min, X-axis segments). The exemplary LDCV of each cell (B, D) is represented within the dotted grey boxes. (**G**) Axial CD size distribution of the two cells shown in (E) and (F) (bin size 10 nm). The overall median value of both cells is 42 nm (gray dotted line). Note the increased contribution of axial CDs smaller than the median as the [Ca^2+^]_i_ was increased from 80 to 650 nM. However, this increase was not as pronounced as that of lateral mobility shown in Figure 2I.

We noticed that the fraction of CDs in the freely mobile state was dramatically reduced to a minimum at 500 nM free Ca^2+^. This represents more than a one-third reduction compared to 100 nM free Ca^2+^ (*P* = 0.037; N = 23 and 26 cells for 100 and 500 nM, respectively, Figure 3B). Furthermore we observed a concomitant increase in the caged and immobile (docked and primed) states at an [Ca^2+^]_i_ of 500 nM compared to 100 nM (Figure 3B). Therefore, docking may be Ca^2+^-sensitive, so we examined the mobility of LDCVs perpendicular to the PM to measure docking. We calculated the relative axial positions (*z_i_*) of each individual vesicle within the evanescent wave based on its fluorescence intensity. The relative changes in LDCV fluorescence over time were further checked with the CD_z_ analysis (Figure 5 same cells as shown in Figure 2). The effects of [Ca^2+^]_i_ on axial mobility were similar to the effects on lateral mobility (Figure 2I), though they were attenuated. The proportion of CDs smaller than the overall median (42 nm) was only slightly increased in the cell kept at a free [Ca^2+^]_i_ of 650 nM compared to the cell maintained at an [Ca^2+^]_i_ of 80 nM (48.6% and 42.9% respectively, Figure 5G).

**Figure 6:**
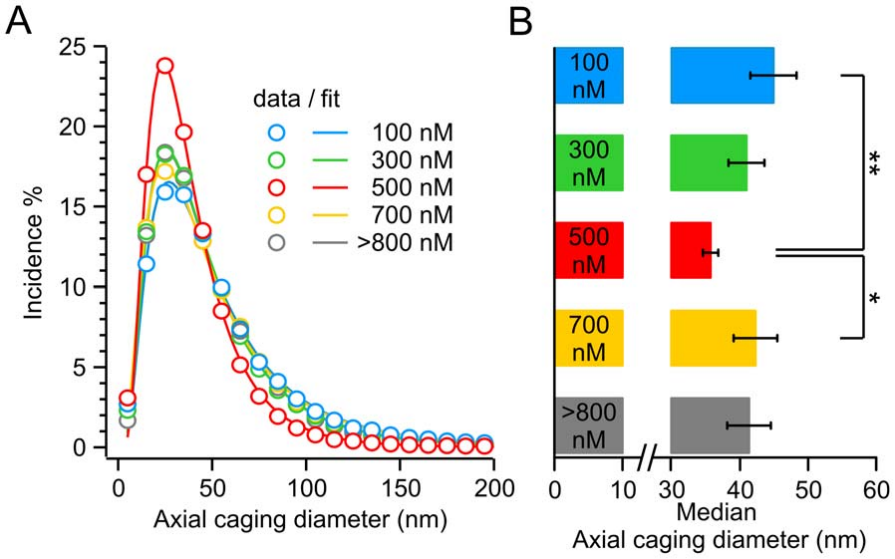
Reduced axial mobility at a free [Ca^2+^]_i_ of 500 nM. (**A**) Average axial CD size distribution for each [Ca^2+^]_i_. The circles represent the averaged data points. The solid lines represent the best fit with a single log-normal component. Note that the peak amplitude at each [Ca^2+^]_i_ is lower than the calculated average amplitude given in the results due to variations in the peak position of the CD histogram of each cell. (**B**) Median of the log-normal fits. Reduction indicates the axial immobilization of LDCVs at 500 nM free Ca^2+^. N_100nM_/n: 23/719; N_300nM_/n: 13/403; N_500nM_/n: 26/695; N_700nM_/n: 14/309; N_>800 nM_/n: 16/390; *P<0.05; **P<0.01.

The average axial CD distribution at various [Ca^2+^]_i_ (Figure 6A, circles; see Figure S3 and Table S3 for full statistical analysis) followed a skewed distribution best fitted by a single log-normal function (Figure 6A, lines). The average peak amplitude was 19.6±1.2% at 100 nM free Ca^2+^ and increased to a maximum of 26.5±1.4% at 500 nM free Ca^2+^ (N = 23 and 26 respectively, *P*<0.001, Figure 6A). Note that, because the peak position of the CD histogram of each cell varies, the peak amplitude at each [Ca^2+^]_i_ shown Figure 6A is lower than the calculated average amplitude given here. In line with the increased peak amplitude, the median decreased significantly from 44.5±2.9 nm at 100 nM Ca^2+^ to a minimum of 34.0±1.3****nm at 500 nM Ca^2+^ (*P* = 0.001, Figure 6B). Thus, the CD distribution shifted left to smaller CD values, i.e. lower mobility at 500 nM Ca^2+^ in comparison to 100 nM. When we further increased the [Ca^2+^]_i,_ we found that this effect was reversed (Figure 6B and Figure S3), revealing that the Ca^2+^-dependence of axial mobility is bell-shaped, with maximal immobilization at 500 nM free Ca^2+^, which is 200 nM lower than the concentration at which maximal lateral immobilization was found to occur. The lateral mobility of LDCVs appears to have a lower sensitivity to free intracellular Ca^2+^ than axial mobility. To illuminate this phenomenon, we plotted each lateral CD with its corresponding axial CD. The slope of the linear regression was reduced at 500 nM [Ca^2+^]_i_ compared to 100 and 700 nM (Figure S4), suggesting that lateral and axial immobilization correspond to two different mechanisms, such as priming and docking.

### LDCVs Showed Little Axial Mobility Immediately Prior to Secretion

**Figure 7:**
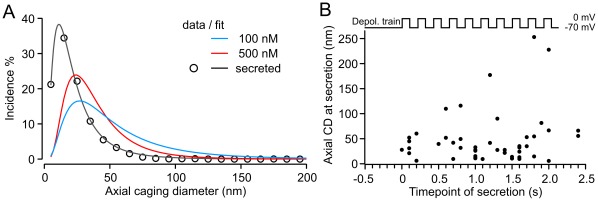
Axial mobility of secreted vesicles was reduced to a minimum immediately before fusion. (**A**) Histogram of the averaged axial CD distribution of secreted vesicles from cells at a pre-stimulus [Ca^2+^]_i_ of 590 nM (black). Data points are represented by circles, and the solid line represents the log-normal fit. The CD values were calculated from the last 15 s prior to fusion induced by a train of depolarizations. The two fits obtained from resting cells kept at 100 nM (blue) and 500 nM (red) [Ca^2+^]_i_ were plotted for comparison. N_100nM_/n: 23/719; N_500nM_/n: 26/695; N_secreted_/n: 19/53. Note the large left shift of the histogram of axial CDs measured from secreted LDCVs compared to the histograms of cells at rest. (**B**) Mobility of LDCVs during the last second prior to secretion. The stimulation protocol is shown on top.

The lateral mobility of LDCVs was strongly diminished just prior to secretion (Figure 4). We verified that axial mobility of the same LDCVs was also reduced by analyzing their axial mobility during the last 15 s before fusion with the PM using the identical algorithm as described above (Figs. 5 and 6). We found that the axial CD histogram of secreted vesicles left-shifted compared to LDCVs in resting cells (Figure 7A). The median value of the axial CD of secreted vesicles was 18.87 nm, nearly two-times smaller than the median axial CD of LDCVs in resting cells maintained at a free [Ca^2+^]_i_ of 500 nM. To assess the degree of axial immobility of vesicles, we measured the mobility of fluorescent beads fixed to a coverslip. Figures 7 and Figure S5 clearly show that the axial CD histogram of secreted LDCVs was more similar to the histogram of fixed beads than the histogram of LDCVs in resting cells. However, the secreted LDCVs were not entirely immobile. Less than one-third of these LDCVs displayed an axial CD value greater than 40 nm (maximal value reached by fixed beads) immediately before fusion with the PM (Figure 7B). LDCVs showing a higher axial mobility in the last second before fusion were secreted at least 0.5 s after the onset of stimulation. These LDCVs experienced a high [Ca^2+^]_i,_ of roughly 10 µM and, therefore, probably undergo immediate docking, priming, and secretion.

### 500 nM of Free [Ca^2+^]_i_ Diminished the Number of LDCVs Visiting the PM

**Figure 8:**
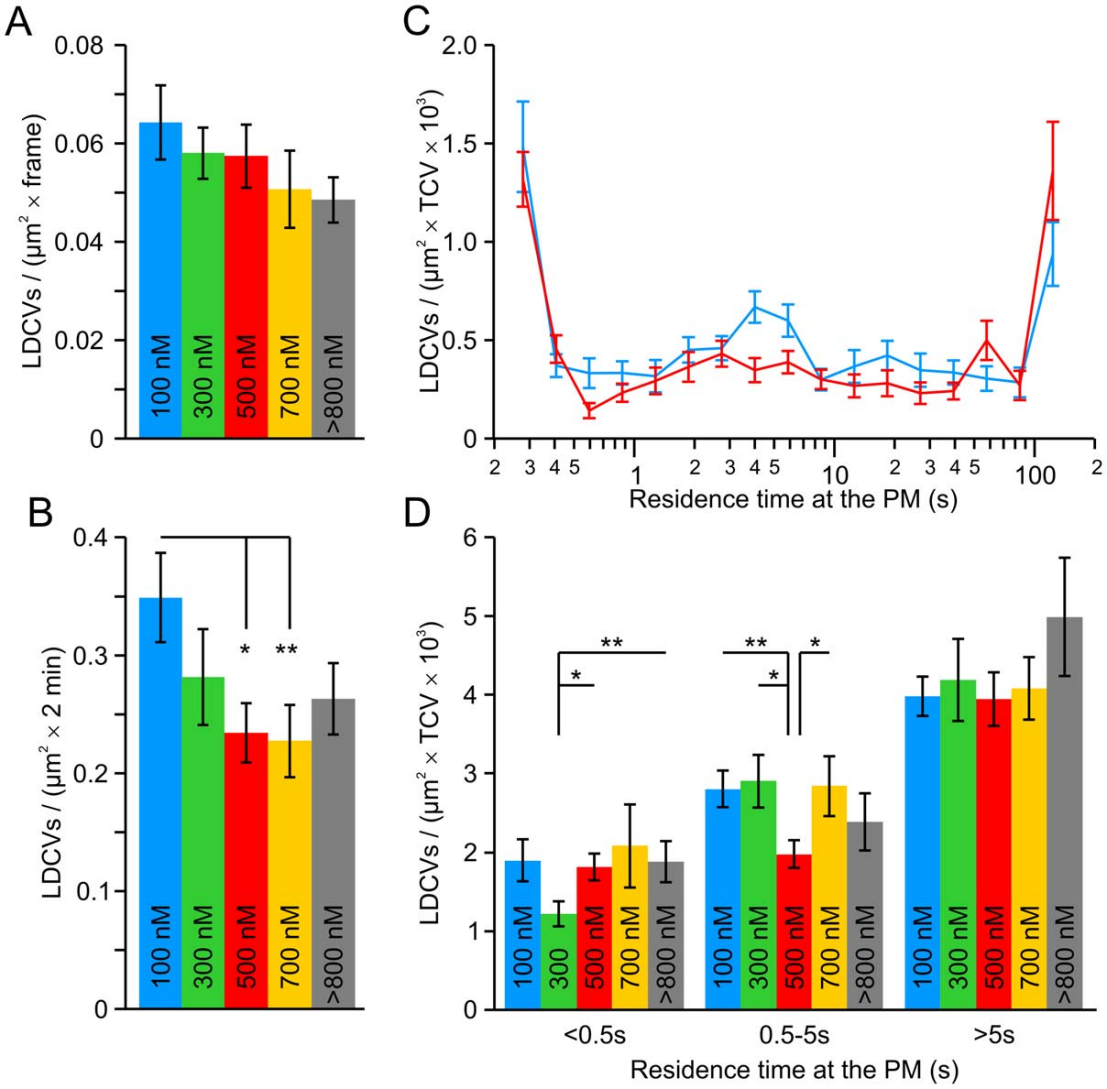
Number of vesicles residing at the plasma membrane between 0.5 and 5 s was reduced at an [Ca^2+^]_i_ of 500 nM compared to 100 nM. (**A**) Density of labeled LDCVs at the footprint of the cell per frame. (**B**) Sum of labeled LDCVs visualized over the whole recording period (2 min) normalized to the footprint area of the cell. A gradual reduction can be observed by increasing the free [Ca^2+^]_i_ above 200 nM. (**C**) Logarithmic plot of vesicles vs. residence time. Data were normalized to the footprint area and the total count of vesicles per cell (TCV) over 2 min (blue: 100 nM [Ca^2+^]_i_; red: 500 nM [Ca^2+^]_i_). (**D**) Averaged distribution of residence times of LDCVs in the evanescent wave at all [Ca^2+^]_i_. Data were normalized to the footprint area and the TCV over 2 min. N_100nM_/n: 23/832; N_300nM_/n: 13/514; N_500nM_/n: 26/958; N_700nM_/n: 14/359; N_>800 nM_/n: 16/512; *P<0.05; **P<0.01.

Because we found that priming and docking may be differentially regulated by [Ca^2+^]_i_, we quantified how long LDCVs remain at the PM. If free intracellular Ca^2+^ is required for docking, then the number of LDCVs at the PM should increase with increasing [Ca^2+^]_i_. However, if [Ca^2+^]_i_ is not an essential docking factor, but has a modulatory role, then the density of LDCVs at the PM would be unaffected by changes in [Ca^2+^]_i_. At [Ca^2+^]_i_ of 100 nM, we counted 0.065±0.008 LDCVs per frame and µm^2^. This number remained roughly constant at all [Ca^2+^]_i_ (100 to >800 nM, Figure 8A). In contrast, the sum of all LDCVs that were visualized during the 2 min recording time was reduced by one-third from 0.35±0.04 LDCVs per µm^2^ at 100 nM free Ca^2+^ to 0.23±0.03 at higher concentrations (*P* = 0.008; Figure 8B). If the density of LDCVs per individual frame remained constant but the number of LDCVs visualized during the entire recording period varies with the [Ca^2+^]_i_ than the time LDCVs reside close to the membrane, i.e. in the evanescent wave, should be Ca^2+^-dependent thereby reflecting a modulation of docking. The residence time for each visualized LDCV was then displayed in a histogram with logarithmically increasing bin sizes. This representation was previously used to successfully assess docking [8]. Figure 8C displays the average result obtained at the two [Ca^2+^]_i_ (100 nM and 500 nM) for which the most striking difference was observed. At 100 nM free Ca^2+^, roughly 10% of the LDCVs resided at the PM for the entire measurement period (2 min). These LDCVs might correspond to a population of LDCVs called dead-end LDCVs, which remain stationary at the PM and do not participate in secretion [1]. As it was not possible to distinguish between functional and dead-end LDCVs, we did not exclude them from the analysis. Roughly 25% of LDCVs could be observed for only a short time at the PM (less than 0.5 s) and were very dim (Figure S1 and Table S1). Thus, these LDCVs were probably more than 200 nm from the PM and most likely not docked. This population was reduced at an [Ca^2+^]_i_ of 300 nM. Finally, approximately two-thirds of the LDCVs remained at the PM for more than 0.5 s and less than 2 min (Figure 8C). Increasing the [Ca^2+^]_i_ significantly reduced the fraction of LDCVs remaining close to the PM between 0.5 and 5 s (Figure 8C, D), a time window which has been associated with ongoing docking [8]. This effect was maximal at 500 nM free [Ca^2+^]_i_ and reverted at higher [Ca^2+^]_i_ (Figure 8D). The obtained result indicates that intracellular Ca^2+^ plays a role in docking whereby a concentration of 500 nM Ca^2+^ appears to have the strongest effect.

## Discussion

In the present work, we examined the role of intracellular Ca^2+^ on LDCV priming and docking in chromaffin cells. We showed that intracellular Ca^2+^ not only modulates docking, but that docking and priming have distinct sensitivities to Ca^2+^; priming was maximal at an [Ca^2+^]_i_ of 700 nM, whereas docking was optimal at an [Ca^2+^]_i_ of 500 nM.

Patch-clamping the cells in whole cell configuration allowed us to maintain the chromaffin cells at a constant and defined [Ca^2+^]_i_, and to assess the effect of various [Ca^2+^]_i_ on priming and docking. Since lateral mobility of LDCVs is related to their functional states in chromaffin cells [7], [9], [17], we simultaneously visualized LDCVs’ mobility over time using TIRF-microscopy. Immobile vesicles have been shown to be primed, whereas docked and unprimed vesicles display a caged mobility [7]. Here, we found that the fraction of immobile vesicles linearly increased with raising [Ca^2+^]_i_, with a maximum lateral immobilization observed at 700 nM of free Ca^2+^. Further increasing the [Ca^2+^]_i_ reverted this effect, which agrees with observations by Allersma et al. [18] showing that LDCVs display a higher mobility at very high Ca^2+^ concentrations (>2 µM) in comparison to resting conditions (i.e. [Ca^2+^]_i_<200 nM) [18]. Priming was also assessed by calculating the pool sizes of the IRP, a subpopulation of the RRP [15]. We found that the size of the IRP increased gradually, reaching a maximum at an [Ca^2+^]_i_ of 700 nM, supporting our results obtained by lateral LDCV mobility analysis.

Both, membrane capacitance measurements and lateral CD analysis, revealed that the pool size of primed LDCVs was maximal at 700 nM of free Ca^2+^. As shown by Voets et al. we cannot exclude that optimal priming conditions for LDCVs occur at higher concentrations [12]. However, above 800 nM [Ca^2+^]_i_ the cells start to secrete consequently docking and priming are not anymore in equilibrium conditions. As a result the pool size of primed LDCVs is decreased which is reflected in an increased LDCV’s mobility. This observation is in full agreement with previous findings [12], [19].

In recent years, the mobility of LDCVs prior to secretion has been a matter of debate. In a previous study, we showed that secreted vesicles are mainly immobile prior to secretion [7]. In the current study, we reproduced and substantiated these results. However, we found that few LDCVs were secreted from the caged mobility state. This occurred as the cells were stimulated for more than 500 ms, at a time point where the [Ca^2+^]_i_ was greater than 10 µM. At this [Ca^2+^]_i_ LDCVs may undergo rapid docking and priming, which would result in one large movement just prior to fusion [20], [21]. Another possibility is that the CD analysis requires a minimum time window of 1 s, and this type of mobility may have been displayed early in the time window but then the vesicles became entirely immobile.

One method that is regularly used to assess docking is to count the number of LDCVs touching the PM in electron micrographs. A diminution of these LDCVs is interpreted as a docking defect. In TIRF-microscopy, a close approximation of this number can be obtained by assessing the LDCV density at the PM. Comparing data obtained by TIRF- and electron microscopy led to an alternative terminology using the word tethering instead of docking and priming [1], [8], [22]. We define docked vesicles as vesicles that are weakly anchored/tethered to the PM, whereas primed vesicles are strongly attached/tethered to the PM through the formation of the soluble NSF attachment protein receptor (SNARE) complex and are thus fusion competent [3]. Here, we observed that increasing [Ca^2+^]_i_ did not affect this density, indicating that Ca^2+^ is not an obligatory docking factor. Instead, the [Ca^2+^]_i_ might have a modulatory role in docking. In line with this argument, we showed that the total number of LDCVs visualized at the PM over 2 min was reduced at an [Ca^2+^]_i_ of 500 nM, which could be explained by a reduced docking rate or increased undocking rate. Previously, the duration of LDCVs remaining close to the PM (i.e. in the evanescent field of TIRF-microscopy) has been used as a complementary method for assessing docking. Toonen et al. [8] showed that deletion of the docking protein Munc18 reduced mainly the number of LDCVs residing at the PM for less than 10 s [8]. In the present study, we showed that increasing [Ca^2+^]_i_ from 100 to 500 nM led to a specific reduction in the pool of LDCVs visiting the PM for a short (0.5 to 5 s) period of time. One interpretation of these data is that high intracellular Ca^2+^ inhibits docking. Several lines of evidence argue against this hypothesis. First, if docking is impeded at higher [Ca^2+^]_i_, the fraction of LDCVs approaching the PM and staying less than 0.5 s in the evanescent field would be reduced by increasing [Ca^2+^]_i_ [8]. In the contrary, we found that LDCVs were not hindered in their approach to the PM and this short living fraction remains unchanged. Second, the cortical actin network is destabilized at high [Ca^2+^]_i_, facilitating the access of LDCVs to the PM [23]. Third, if high Ca^2+^ reduces docking, then LDCVs would only approach the PM and shortly thereafter move away, resulting in large axial mobility. We observed the opposite; the axial mobility was reduced to a minimum at an [Ca^2+^]_i_ of 500 nM, showing the same Ca^2+^-dependency as the residence time of LDCVs at the PM. The alternative interpretation of our data is that Ca^2+^ modulates docking efficiency by reducing the undocking rate. If this conclusion is true, then in cells in which docking and priming are in equilibrium increasing the [Ca^2+^]_i_ would stabilize the docked pool of LDCVs, thereby reducing the turnover rate of LDCVs at the PM. This is precisely what we observed in cells that did not secrete, i.e. cells maintained at [Ca^2+^]_i_ up to 700 nM (Figure 1C). When the [Ca^2+^]_i_ is raised to higher concentrations, secretion is induced, the functional pools are not anymore in equilibrium and the net docking and priming rates are altered. De Wit et al. [24] suggested that docking and priming sites are identical because the acceptor proteins on the PM (Syntaxin1 and SNAP-25) appear to be involved in both processes ([24]; see also [25]). Therefore, raising [Ca^2+^]_i_ from 500 to 700 nM should lead to a simple redistribution of the LDCVs from the docked into the primed pool. Our observations concur with this prediction, confirming that Ca^2+^ modulates docking efficiency by reducing the undocking rate.

Interestingly, axial mobility displayed only a single component, whereas lateral mobility comprised three. We speculate that, after docking, the following priming step of LDCVs does not result in additional axial immobilization. Furthermore LDCVs that are neither primed nor docked likely remain far from the PM, at the fringe of the evanescent wave. These vesicles were characterized by a free lateral mobility state and a short residence time at the PM. Although such vesicles would display large axial movement, they produce few CD_z_ values, and their influence on the axial CD distribution is negligible.

Our results show that lateral and axial mobility can be used as precise readouts for priming and docking, respectively. The question that arises is whether these small but significant differences in the Ca^2+^-sensitivity of docking and priming are physiological relevant. Modeling [Ca^2+^]_i_ gradients close to the PM that arise upon stimulation, Klingauf and Neher [26] found that with a Ca^2+^ channel density of roughly 15/µm^2^ (as found in chromaffin cells), the Ca^2+^ spread is different in lateral and axial directions due to the lateral overlap of Ca^2+^ microdomains. At a distance of 150 nm from the channels, the [Ca^2+^]_i_ is >300 nM along the PM and approximately 240 nM perpendicular to the PM (see Figure 4B in [26]). Thus, during basal stimulation at 0.5 Hz [27], as occurs in feed and breed responses, vesicles that are in the process of docking, i.e. moving towards the PM, are exposed to a steep Ca^2+^ gradient. Already primed vesicles are surrounded by a relatively high basal [Ca^2+^]_i_. Therefore, promoting docking to the same extent as priming would require a slightly different Ca^2+^-sensitivity. Using this mechanism, primed and docked pools are filled to an optimum in the feed and breed mode, which allows a maximal response in the case of a fight or flight stimulus (stimulation frequency 15 Hz; [28]).

Overall our results point to intriguing implications about the molecular mechanism underlying this differential modulation of docking and priming. Several putative proteins have been suggested to mediate the Ca^2+^-sensitivity of priming. The principal candidates are Munc13 and CAPS, known priming factors both contain C2 domains with low Ca^2+^-sensitivity (Kd >5 µM). Munc13 also contains an active calmodulin binding site [29], [30], [31]. Yet no protein has been identified as a Ca^2+^-sensor for priming. The most promising candidate for such a sensor is DOC2, a double C2 domain protein [17] that interacts with Munc13 in a Ca^2+^-dependent manner (Kd ≈ 300 nM) and appears to facilitate priming [32], [33]. The Ca^2+^-sensor for docking is even more elusive, with only two identified docking factors. Recently de Wit et al. [24] showed that the interaction of synaptotagmin with the t-SNAREs Syntaxin1 and SNAP25 is required for LDCV docking in chromaffin cells, but didn’t demonstrate if there was Ca^2+^-dependence. Whereas Toonen et al. [8] showed that Munc18-1 interacts with actin, disrupting the thick actin cortex and allowing LDCVs to approach the PM [8]. However, Munc18-1 has no known Ca^2+^-binding site. Interestingly, the actin disruption, which appears to be a major step that enables docking. is not only mediated by Munc18, but also by scinderin [34], [35]. In contrast to all other aforementioned proteins, scinderin has a Ca^2+^ binding site with a Kd of 585 nM, which would be compatible with our estimation of the Ca^2+^-sensitivity of docking.

In conclusion, examining both the lateral and axial mobility of LDCVs in the evanescent wave enabled us to distinguish between docked and primed vesicles, which we showed to occur at different [Ca^2+^]_i_. This provides a framework in which the factors involved in these Ca^2+^-dependent reactions can now be identified.

## Materials and Methods

### Cell Preparation and Transfection

Isolated bovine adrenal chromaffin cells were obtained from the slaughterhouse (Emil Färber GmbH & Co. KG Zweibrücken, Germany) and prepared as described previously [7]. LDCVs were labeled by over-expressing NPY fused to mCherry [36]. For most experiments, we used the Semliki Forest Virus system [7]. We transfected the cells via electroporation (Gene pulser II, Biorad, Hercules, CA, USA) for experiments designed to measure the mobility of LDCVs prior to secretion.

### Solutions

The bath solutions contained (in mM): 145 NaCl, 2.4 KCl, 1 CaCl_2_, 4 MgCl_2_, 10 HEPES, and 10 glucose. We used a low [CaCl_2_] and high [MgCl_2_] to reduce Ca^2+^-influx and obtain a constant [Ca^2+^]_i_ over a long period of time (∼5 min). In the experiments in which we measured the mobility of LDCVs prior to fusion, we changed the [Ca^2+^] and [MgCl_2_] to 2.5 and 1 mM. All other components of the solution remained the same. The bath solutions were at pH 7.4, 315 mOsm. The three different intracellular solutions for patch-clamping experiments are shown Table 1. These solutions were designed to have nearly equal and a relatively low Ca^2+^-buffer capacity in order to minimize perturbance of endogenous Ca^2+^-buffers and to enable exocytosis upon depolarization. Free EGTA and Ca^2+^-indicator concentrations were calculated using an IGOR macro (Freecon; Wave Metrics, Portland, OR, USA). Osmolarity was adjusted with Cs-glutamic-acid to a value of 300 mOsm (pH 7.25). We used a combination of Fura2 (K_d_(Ca^2+^) ∼140 nM) and FuraFF (K_d_(Ca^2+^) ∼5.5 µM; both Invitrogen, Carlsbad, CA, USA) to measure the [Ca^2+^]_i_ with high precision in a range of 0–10 µM. All chemicals were obtained from Sigma (Steinsheim, Germany).

An in vivo calibration curve was used to convert the ratio of fluorescent signals at both wavelengths (350 and 380 nm) into the [Ca^2+^]_i_. Due to variations in the inherent Ca^2+^-buffer capacity of the cell, using only three different intracellular solutions allowed us to impose a wide range of [Ca^2+^]_i_: 70–220 nM with solution 1, 170–450 nM with solution 2, and 450–2000 nM with solution 3.

### Membrane Capacitance Recordings

Whole-cell recordings were performed with 3–5 MOhm patch pipettes using an EPC-9 patch-clamp amplifier controlled by PULSE software (Heka Elektronik, Lambrecht, Germany). Cells were maintained at a holding potential of -70 mV. Capacitance measurements were carried out with the Lindau-Neher technique implemented as the “sine + dc” mode of the “software lock-in” extension of the PULSE software. A 1 kHz, 70 mV peak-to-peak sinusoid stimulus was applied about a DC holding potential of −70 mV. Data were acquired through a combination of the high time resolution PULSE software (2 kHz) and the lower time resolution X-Chart plug-in module to the PULSE software at 1 Hz. After imaging, exocytosis was induced by a train of depolarizations to 0 mV composed of four short (10 ms, 6 Hz) and three long (100 ms, 5 Hz) stimuli at 100-ms intervals. This stimulation protocol allowed us to measure the size of the IRP [15]. In the experiments in which we followed the mobility of LDCVs prior to secretion, we stimulated the cells with a train of ten depolarizations of 100 ms at 5 Hz. To maintain comparable Ca^2+^ influx, the amplitudes of the following depolarizing pulses were adjusted, i.e. applied voltage was gradually increased over the stimulus period from -5 mV to +10 mV.

### Calculation of IRP Size

The upper limits of the IRPs were calculated using the formula from Voets et al. ([15]; see also [37]). Calculations are based on the relationship between the capacitance changes (ΔC_m_) in the first two 10-ms depolarizations (Eq. 1) [37].
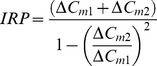
(1)Only cells that exhibited a ratio of <0.7 were used for analysis, as substantial pool depletion is a prerequisite for accurate pool size determination. In our measurements, more than 95% of all cells in which the [Ca^2+^]_i_ was <800 nM prior to stimulation fulfilled this condition. Cells that were maintained at higher [Ca^2+^]_i_ did not fulfill this condition, and the calculation only approximates their IRP size.

### TIRF-microscopy

TIRF-microscopy was carried out as described by Becherer et al. [14]. The setup is based on an inverted Zeiss (Göttingen, Germany) Axiovert 200 with a Zeiss TIRF-slider and a solid-state laser system (85YCA010; Melles Griot, Carlsbad, CA) emitting at 561 nm. The filter sets contained an UV-reflecting dual-band dichroic mirror (catalog #F53–563; AHF Analysentechnik, Tübingen, Germany) and an emission filter (catalog #F72–419). The objective used for all measurements was the 100×/1.45 NA Fluar (Zeiss). The setup was equipped with an EMCCD camera (Andor iXon^EM^, Belfast, Northern Ireland) and controlled, like the rest of the setup, by in-house software based on LabView (National Instruments, München, Germany). Final pixel size was 160 nm. The acquisition rate was 10 Hz with an exposure time of 75 ms. Additionally, a TILL Polychrome V monochromator (TILL-Photonics, Gräfelfing, Germany) was mounted at the rear port of the microscope via a light guide to ratiometrically measure the [Ca^2+^]_i_ (excitation wavelengths 350 and 380 nm, emission 510 nm).

### Image Recording Protocol

In order to obtain stable and consistent measurements, cells were imaged after a loading period of 2 min without exciting the fluorescently labeled NPY. During this loading period, the [Ca^2+^]_i_ was determined every 2.5 s. Only cells with a constant [Ca^2+^]_i_ for more than 30 s before the end of the loading procedure were used for experiments. After LDCV imaging (2 min at 10 Hz), the [Ca^2+^]_i_ was determined again to verify that it was similar to the concentration measured at the end of the loading process. After an additional minute of recording, the cell was stimulated as described above to confirm the viability of the cell, its ability to secrete, and to measure the IRP size. In the experiments in which we measured secretion simultaneously with TIRF-microscopy and patch-clamping, the [Ca^2+^]_i_ was continuously measured at 0.4 Hz throughout the experiment. The secretion was induced 10 s before the end of the 2-min imaging period. All measurements were performed at room temperature.

### Tracking Procedure and Mobility Analysis

The vesicles were tracked using software developed with LabView (LV, National Instruments, Munich, Germany). First, the coarse position of a vesicle was determined by a LV routine that calculates the center of mass of each object in the binarized images. Then the localisation of the LDCVs was refined by calculating the centroid position (Eqs. 2, 3) of a 5×5 region of interest drawn around the XY center of mass of each vesicle on the raw image. Thus, precise subpixel accuracy was achieved.
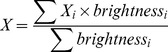
(2)

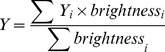
(3)No image processing was performed other than local background subtraction. The threshold for detection was such that vesicles with low fluorescence could be detected; the fluorescent intensity of bright vesicles was higher than 15 times the s.d. of the background noise. The weakest stained LDCVs had a fluorescent intensity only 4 times above the s.d. When fluorescence intensity of a vesicle decreased below the threshold of detection for less than 1.5 s but could still be detected by eye, the trajectories of the vesicles were concatenated. Missing positions were approximated using an interpolating algorithm and then refined using the centroid method. Fluorescent beads (FluoSphere, Ø = 200 nm; Invitrogen, Carlsbad, CA, USA) were used to assess complete immobility. These beads were permanently fixed on a glass-coverslip and imaged at 10 Hz for 1 min at reduced laser intensity in order to mimic the fluorescent intensity of fluorescently labeled LDCVs.

Vesicle mobility was analyzed using CD analysis [7]. For each trajectory, a sliding window of 1 s was set in which the distance between the 1st position occupied by the vesicle and all the other positions within the 1 s window were measured and the maximum distance reached (CD) was determined. Thus, if a vesicle was observed for 2 min (1200 frames), then we obtained 1190 CD values for this particular vesicle (1200–10, 10 = length of time window). Determining an accurate XY position based on a centroid algorithm instead of the center of mass method, which is based on binarized images, improved the signal-to-noise ratio of the CD measurements and enabled us to reduce the size of the time window of the CD analysis from 6 s, as used previously, to 1 s [7], [11]. This change did not affect the overall outcome of the CD analysis, but it enabled us to uncover fine differences in the behavior of LDCVs. In particular, small movements were underestimated when a time window of 6 s was applied. CD analysis was performed for lateral and axial movement. The lateral movements were obtained from the (x, y) coordinates of the LDCVs, whereas the axial movements were inferred from the fluorescent intensity of the LDCVs in each frame.

As described above, the CD method is based on distances. The one-dimensional CD in the z direction (CD_z_) of a vesicle in image *i* is defined by Eq. (4).
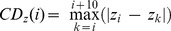
(4)

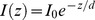
(5)where *I_0_* represents the intensity at distance 0 from the interface and *d* the penetration depth of the evanescent wave [38]. Thus, *z* is defined by Eq. (6).



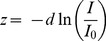
(6)Therefore, Eq. (4) is equivalent to Eq. (7).
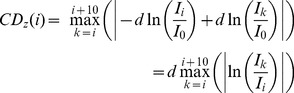
(7)


Fortunately, *I_0_*, which is strongly dependent on the content of fluorophores, does not appear in this relationship. The experimental *d* was 226±18 nm (s.d., n = 6) and determined using a 1-µm fluorescent bead (Invitrogen, Carlsbad, CA, USA) as described by Keyel et al. ([39]; see also [40]). Thus, *d* varied from one recording to another by roughly 8% due to slight variations in the TIRF-angle or irregularities in the glass coverslip. These variations did not introduce any systematic error because the [Ca^2+^]_i_ applied to the cell was chosen randomly. Finally, bleaching of mCherry was minimal in our measurement conditions (less than 10% over 2 min). Furthermore, the axial CD does not depend on the fluorescent intensity of the vesicle at the beginning of the imaging period (I at t = 0 s). Each CD value was calculated using the fluorescent intensity of the vesicle measured in the first image of the sliding time window (I at t = i; see Eq. 6), which is only 1 s. Thus, we assessed changes in axial mobility with great accuracy.

CD measurements and analysis were carried out in IGOR (Wavemetrics, Lake Oswego, OR).

### Fitting of Histograms

We applied a weighted fit procedure using the inverse SD of the average data at each [Ca^2+^]_i_. The lateral CD histogram was best fitted by the sum of three components, namely one log-normal and two Gaussian functions (Eq. 8).
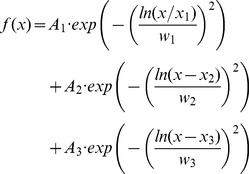
(8)Where *A_n_* is the amplitude of each peak, *x_n_* describes the peak position, and *w_n_* describes its half width (1–3 are the different parameters for the three components). *w_1_* is the half width of the log-normal component, it has no unit, and is related to the variance σ by *w_1_* =  σ·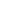 (see Eq. 9 and 9a). Two other models were tested: either a triple Gauss or a double log-normal. Both fits with three components had the same degree of freedom. Thus, the triple Gauss could be rejected due to doubling of the χ^2^ values. Because the CD analysis results in a large fraction of very small and only positive values, the CD distribution is skewed and best described by a log-normal curve [16]. However, the distribution of larger CD values does not fulfill the aforementioned criteria and was fitted by two Gaussian curves. The double log-normal fit was compared with the more complicated description by one log-normal and two Gaussian curves using an F test (Eq. 9).
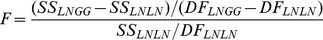
(9)Where SS is the sum of squares, DF the degree of freedom, LNGG the one log-normal and two Gaussian components, and LNLN the two log-normal components. At 500 and 700 nM free Ca2+, the F ratio was 10.2 and 3.1, respectively. Thus, the more complicated model (one log-normal and two Gaussian curves) was preferred.

The axial CD histogram was best fitted with a simple log-normal distribution (Eq. 10). None of the other models (simple or double Gaussian) provided better results (data not shown).
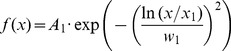
(10)where *A_1_* is the amplitude, *x_1_* the peak position, and *w_1_* represents the width. This equation is equivalent to Eq. (10a).
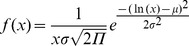
(10a)where μ and σ are the mean and standard deviation of the variable’s natural logarithm.

### Statistical Analysis

Normally distributed data were analyzed using the two-way ANOVA followed by post-tests using Holm-Sidak corrections. For non-normally distributed data, an ANOVA on ranks was used in SigmaPlot (Systat Software, Port Richmond, CA). Results are shown as mean±s.e.m. unless otherwise noted. Numbers of measured cells and vesicles were give as N and n, respectively.

## Supporting Information

Figure S1 **LDCVs residing shortly in the evanescent wave were dim thus presumably at some distance from the plasma membrane.** In order to verify that vesicles residing shortly at the plasma membrane were probably not docked we plotted the median fluorescence intensity of each LDCV against its residence time. The median fluorescence intensity of the LDCVs was normalized to the brightest LDCV in the cell to account for variations in the expression level of NPY-mCherry in each cell. The vertical black lines correspond to the time bins used Figure 6D. The horizontal lines correspond to arbitrary boarder used to statistically analyze this plot (Table S1). We plotted two randomly chosen cells maintained at each [Ca^2+^]_i_ (blue circles, 100 nM; green circles, 300 nM; red circles, 500 nM; orange circles, 700 nM; gray circles, >800 nM).
(TIF)

Figure S2 **Lateral CD frequency distribution with SEM.** blue circles, 100 nM; green circles, 300 nM; red circles, 500 nM; orange circles, 700 nM; gray circles, >800 nM [Ca^2+^]_i_).
(TIF)

Figure S3 **Axial CD frequency distribution with SEM**. blue circles, 100 nM; green circles, 300 nM; red circles, 500 nM; orange circles, 700 nM; gray circles, >800 nM [Ca^2+^]_i_).
(TIF)

Figure S4 **Lateral vs. axial CD correlation plot.** (A, B, C) To analyze the correlation of lateral and axial mobility, the CD of each LDCV which was used for the analysis of the axial movements was plotted versus its lateral CD on a double-logarithmic scale: (A) 100 nM, (B) 500 nM and (C) 700 nM. A large number of points overlap. To visualize this, the density of the CDs is color encoded. The more points are present on one spot the darker becomes this spot. The plots were fitted by a linear curve (f(x) = a+bx) and the slope b is shown on each curve. Note that this slope was specifically reduced at 500 nM free Ca^2+^. This indicates that the mobility of LDCVs from and to the PM is decreased at lower [Ca^2+^]_i_ than their mobility along the PM (blue circles, 100 nM; red circles, 500 nM; orange circles, 700 nM [Ca^2+^]_i_).
(TIF)

Figure S5 **Comparison between lateral or axial CD histogram of secreted vesicles and fixed fluorescent beads.** (A) Lateral CD histogram of secreted vesicles (solid line) and fixed beads (dashed line). (B) Axial CD histogram of secreted vesicles (solid line) and fixed beads (dashed line). Note that in contrast to the lateral histogram the shape of the axial CD histogram of secreted vesicles is similar to the shape of the axial histogram of fixed beads.
(TIF)

Table S1 **Statistical analysis of the fluorescence intensity vs. residence time correlation plot.** RT stands for residence time; NMFI stands for normalized median fluorescence intensity.
(DOC)

Table S2 **Statistical analysis over lateral CD distribution.** P value obtained through two way ANOVA on Rank using Holm-Sidak post-test.100 nM: N = 23, n = 719; 300 nM: N = 13, n = 403; 500 nM: N = 26, n = 695; 700 nM N = 14, n = 309; >800 nM: N = 16, n = 452.
(DOC)

Table S3 **Statistical analysis over axial CD distribution**. P value obtained through two way ANOVA on Rank using Holm-Sidak post-test. 100 nM: N = 23, n = 719; 300 nM: N = 13, n = 403; 500 nM: N = 26, n = 695; 700 nM N = 14, n = 309; >800 nM: N = 16, n = 452.
(DOC)

Movie S1 **Bovine chromaffin cell perfused with [Ca2+] at 80 nM.** Recording of the representative bovine chromaffin cells transfected with plasmid expressing NPY-mCherry kept at stable [Ca2+]i of ∼80 nM (see Fig. 3A). LDCVs are highly mobile and the number of vesicles with short residence times is high. Recording speed: 10 Hz; Recording period: 2 min; Display: 50Hz; Scale bar: 2 µm.
(MOV)

Movie S2 **Bovine chromaffin cell perfused with [Ca2+] at 650 nM.** Recording of the representative bovine chromaffin cells transfected with plasmid expressing NPY-mCherry kept at stable [Ca2+]i of ∼650 nM (see Fig. 3A). LDCVs are very immobile and nearly no visitors can be observed. Recording speed: 10 Hz; Recording period: 2 min; Display: 50Hz; Scale bar: 2 µm.
(MOV)

## References

[1] Verhage M, Sorensen JB (2008). Vesicle docking in regulated exocytosis.. Traffic.

[2] Plattner H, Artalejo AR, Neher E (1997). Ultrastructural organization of bovine chromaffin cell cortex-analysis by cryofixation and morphometry of aspects pertinent to exocytosis.. J Cell Biol.

[3] Becherer U, Rettig J (2006). Vesicle pools, docking, priming, and release.. Cell Tissue Res.

[4] Steyer JA, Horstmann H, Almers W (1997). Transport, docking and exocytosis of single secretory granules in live chromaffin cells.. Nature.

[5] Oheim M, Loerke D, Stuhmer W, Chow RH (1998). The last few milliseconds in the life of a secretory granule. Docking, dynamics and fusion visualized by total internal reflection fluorescence microscopy (TIRFM).. Eur Biophys J.

[6] Johns LM, Levitan ES, Shelden EA, Holz RW, Axelrod D (2001). Restriction of secretory granule motion near the plasma membrane of chromaffin cells.. J Cell Biol.

[7] Nofal S, Becherer U, Hof D, Matti U, Rettig J (2007). Primed vesicles can be distinguished from docked vesicles by analyzing their mobility.. J Neurosci.

[8] Toonen RF, Kochubey O, de Wit H, Gulyas-Kovacs A, Konijnenburg B (2006). Dissecting docking and tethering of secretory vesicles at the target membrane.. Embo J.

[9] Yizhar O, Ashery U (2008). Modulating vesicle priming reveals that vesicle immobilization is necessary but not sufficient for fusion-competence.. PLoS ONE.

[10] Neher E, Zucker RS (1993). Multiple calcium-dependent processes related to secretion in bovine chromaffin cells.. Neuron.

[11] Neher E, Sakaba T (2008). Multiple roles of calcium ions in the regulation of neurotransmitter release.. Neuron.

[12] Voets T (2000). Dissection of three Ca^2+^-dependent steps leading to secretion in chromaffin cells from mouse adrenal slices.. Neuron.

[13] von Ruden L, Neher E (1993). A Ca-dependent early step in the release of catecholamines from adrenal chromaffin cells.. Science.

[14] Becherer U, Pasche M, Nofal S, Hof D, Matti U (2007). Quantifying exocytosis by combination of membrane capacitance measurements and total internal reflection fluorescence microscopy in chromaffin cells.. PLoS ONE.

[15] Voets T, Neher E, Moser T (1999). Mechanisms underlying phasic and sustained secretion in chromaffin cells from mouse adrenal slices.. Neuron.

[16] Limpert E, Stahel W, Abbt M (2005). Log-normal Distributions across the Sciences: Keys and Clues.. BioScience.

[17] Friedrich R, Groffen AJ, Connell E, van Weering JR, Gutman O (2008). DOC2B acts as a calcium switch and enhances vesicle fusion.. J Neurosci.

[18] Allersma MW, Bittner MA, Axelrod D, Holz RW (2006). Motion matters: secretory granule motion adjacent to the plasma membrane and exocytosis.. Mol Biol Cell.

[19] Soerensen JB, Matti U, Wei SH, Nehring RB, Voets T (2002). The SNARE protein SNAP-25 is linked to fast calcium triggering of exocytosis.. Proc Natl Acad Sci U S A.

[20] Degtyar VE, Allersma MW, Axelrod D, Holz RW (2007). Increased motion and travel, rather than stable docking, characterize the last moments before secretory granule fusion.. Proc Natl Acad Sci U S A.

[21] Karatekin E, Tran VS, Huet S, Fanget I, Cribier S (2008). A 20-nm step toward the cell membrane preceding exocytosis may correspond to docking of tethered granules.. Biophys J.

[22] Gulyas-Kovacs A, de Wit H, Milosevic I, Kochubey O, Toonen R (2007). Munc18–1: sequential interactions with the fusion machinery stimulate vesicle docking and priming.. J Neurosci.

[23] Trifaro JM, Gasman S, Gutierrez LM (2008). Cytoskeletal control of vesicle transport and exocytosis in chromaffin cells.. Acta Physiol (Oxf).

[24] de Wit H, Walter AM, Milosevic I, Gulyas-Kovacs A, Riedel D (2009). Synaptotagmin-1 docks secretory vesicles to syntaxin-1/SNAP-25 acceptor complexes.. Cell.

[25] Young SM, Neher E (2009). Synaptotagmin has an essential function in synaptic vesicle positioning for synchronous release in addition to its role as a calcium sensor.. Neuron.

[26] Klingauf J, Neher E (1997). Modeling buffered Ca2+ diffusion near the membrane: implications for secretion in neuroendocrine cells.. Biophys J.

[27] Brandt BL, Hagiwara S, Kidokoro Y, Miyazaki S (1976). Action potentials in the rat chromaffin cell and effects of acetylcholine.. J Physiol.

[28] Fulop T, Radabaugh S, Smith C (2005). Activity-dependent differential transmitter release in mouse adrenal chromaffin cells.. J Neurosci.

[29] Ashery U, Varoqueaux F, Voets T, Betz A, Thakur P (2000). Munc13–1 acts as a priming factor for large dense-core vesicles in bovine chromaffin cells.. Embo J.

[30] Stevens DR, Rettig J (2009). The Ca(2+)-dependent activator protein for secretion CAPS: do I dock or do I prime?. Mol Neurobiol.

[31] Junge HJ, Rhee JS, Jahn O, Varoqueaux F, Spiess J (2004). Calmodulin and Munc13 form a Ca^2+^ sensor/effector complex that controls short-term synaptic plasticity.. Cell.

[32] Duncan RR, Betz A, Shipston MJ, Brose N, Chow RH (1999). Transient, phorbol ester-induced DOC2-Munc13 interactions in vivo.. J Biol Chem.

[33] Orita S, Naito A, Sakaguchi G, Maeda M, Igarashi H (1997). Physical and functional interactions of Doc2 and Munc13 in Ca^2+^-dependent exocytotic machinery.. J Biol Chem.

[34] Dumitrescu Pene T, Rose SD, Lejen T, Marcu MG, Trifaro JM (2005). Expression of various scinderin domains in chromaffin cells indicates that this protein acts as a molecular switch in the control of actin filament dynamics and exocytosis.. J Neurochem.

[35] Rodriguez Del Castillo A, Lemaire S, Tchakarov L, Jeyapragasan M, Doucet JP (1990). Chromaffin cell scinderin, a novel calcium-dependent actin filament-severing protein.. Embo J.

[36] Shaner NC, Campbell RE, Steinbach PA, Giepmans BN, Palmer AE (2004). Improved monomeric red, orange and yellow fluorescent proteins derived from Discosoma sp. red fluorescent protein.. Nat Biotechnol.

[37] Gillis KD, Mossner R, Neher E (1996). Protein kinase C enhances exocytosis from chromaffin cells by increasing the size of the readily releasable pool of secretory granules.. Neuron.

[38] Axelrod D (2001). Total internal reflection fluorescence microscopy in cell biology.. Traffic.

[39] Keyel PA, Watkins SC, Traub LM (2004). Endocytic adaptor molecules reveal an endosomal population of clathrin by total internal reflection fluorescence microscopy.. J Biol Chem.

[40] Fish KN (2009). Total internal reflection fluorescence (TIRF) microscopy.. Curr Protocol Cytom.

