# Supplementary figures and images for: Docking of LDCVs Is Modulated by Lower Intracellular [Ca2+] than Priming
Source: PLoS One. 2012 May 10;7(5):e36416. doi: 10.1371/journal.pone.0036416 (PMC3349663; doi:10.1371/journal.pone.0036416)

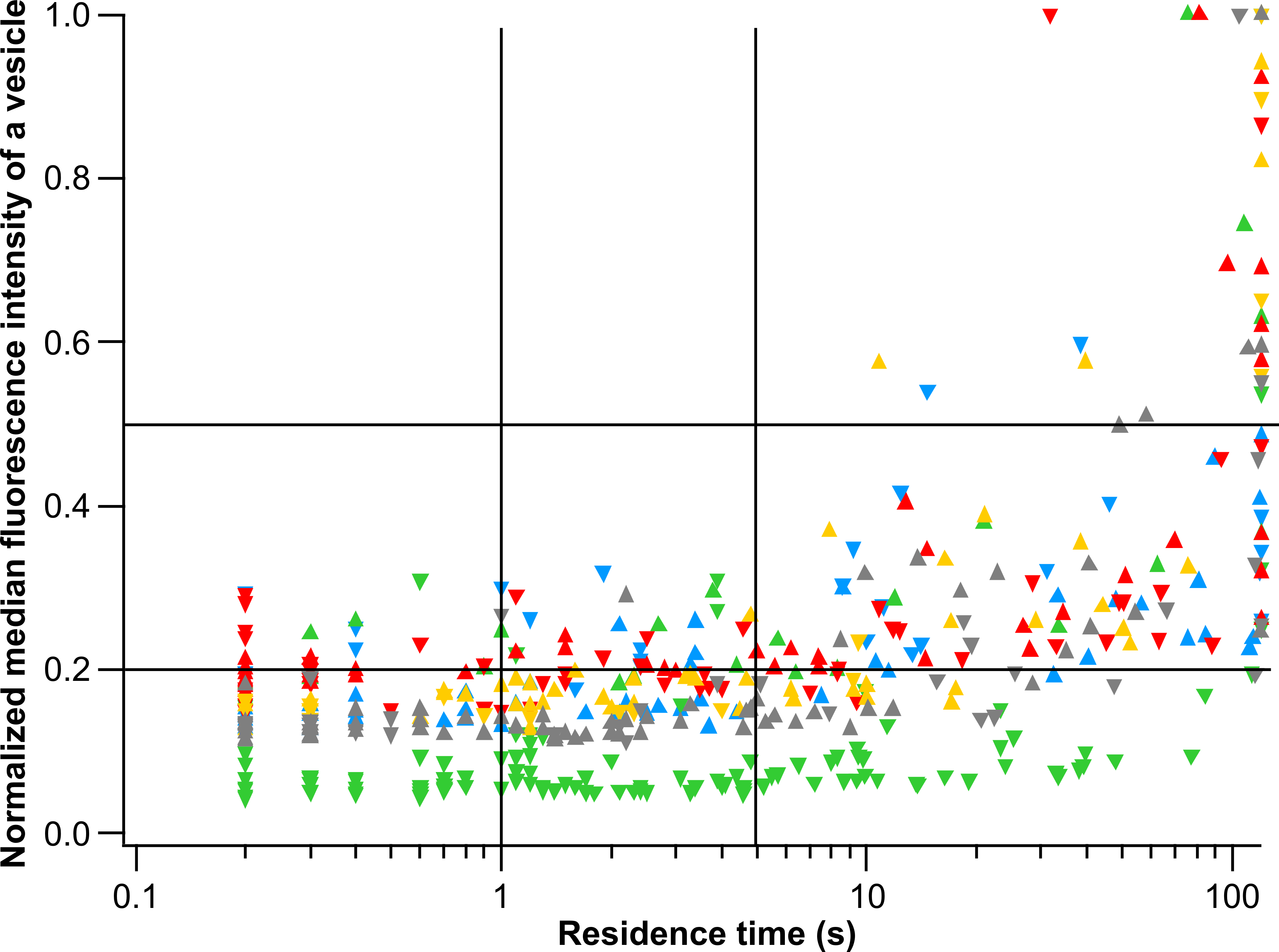

Supplement: Figure S1 — LDCVs residing shortly in the evanescent wave were dim thus presumably at some distance from the plasma membrane. In order to verify that vesicles residing shortly at the plasma membrane were probably not docked we plotted the median fluorescence intensity of each LDCV against its residence time. The median fluorescence intensity of the LDCVs was normalized to the brightest LDCV in the cell to account for variations in the expression level of NPY-mCherry in each cell. The vertical black lines correspond to the time bins used Figure 6D. The horizontal lines correspond to arbitrary boarder used to statistically analyze this plot (Table S1). We plotted two randomly chosen cells maintained at each [Ca2+]i (blue circles, 100 nM; green circles, 300 nM; red circles, 500 nM; orange circles, 700 nM; gray circles, >800 nM). (TIF) [file pone.0036416.s001.tif]

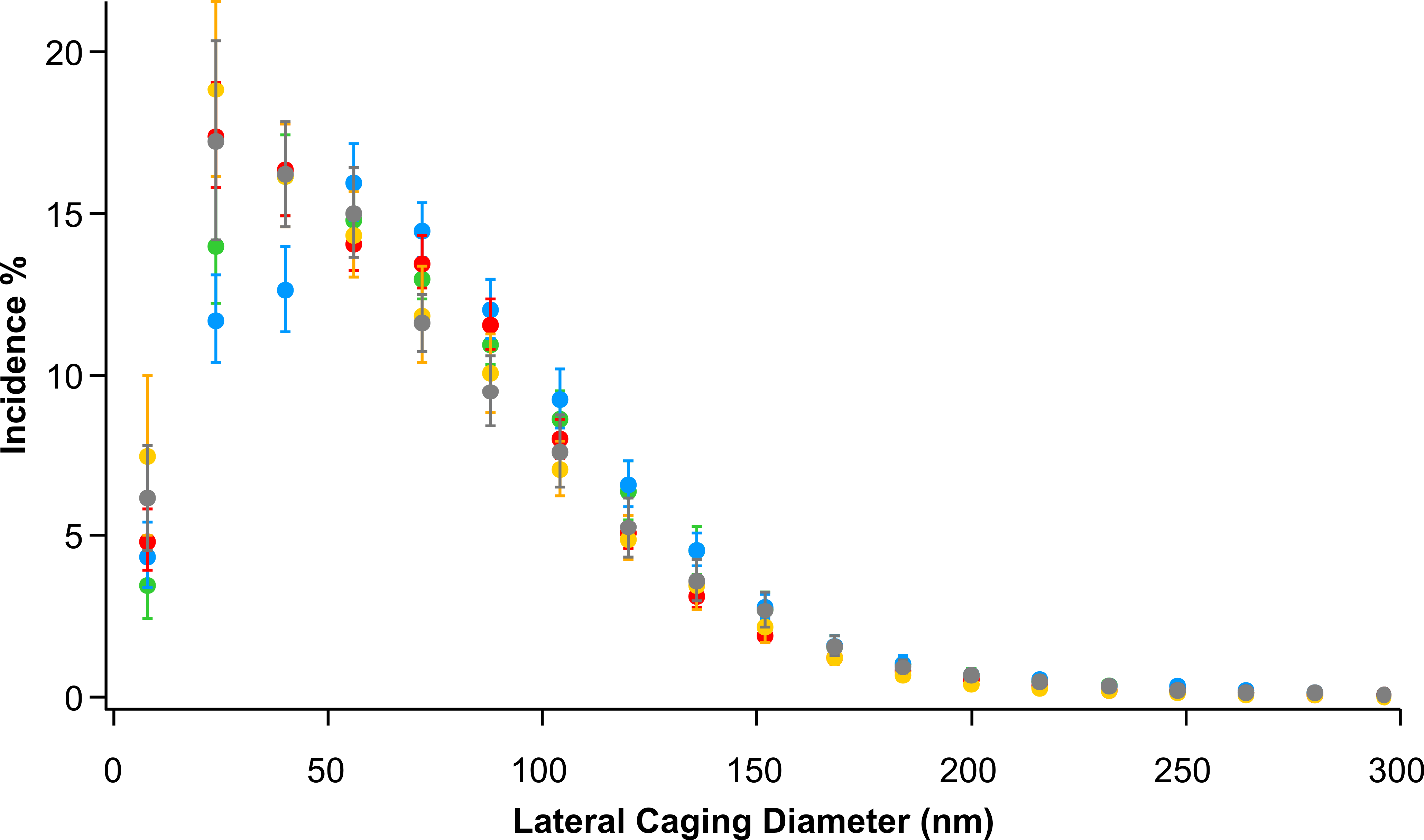

Supplement: Figure S2 — Lateral CD frequency distribution with SEM. blue circles, 100 nM; green circles, 300 nM; red circles, 500 nM; orange circles, 700 nM; gray circles, >800 nM [Ca2+]i). (TIF) [file pone.0036416.s002.tif]

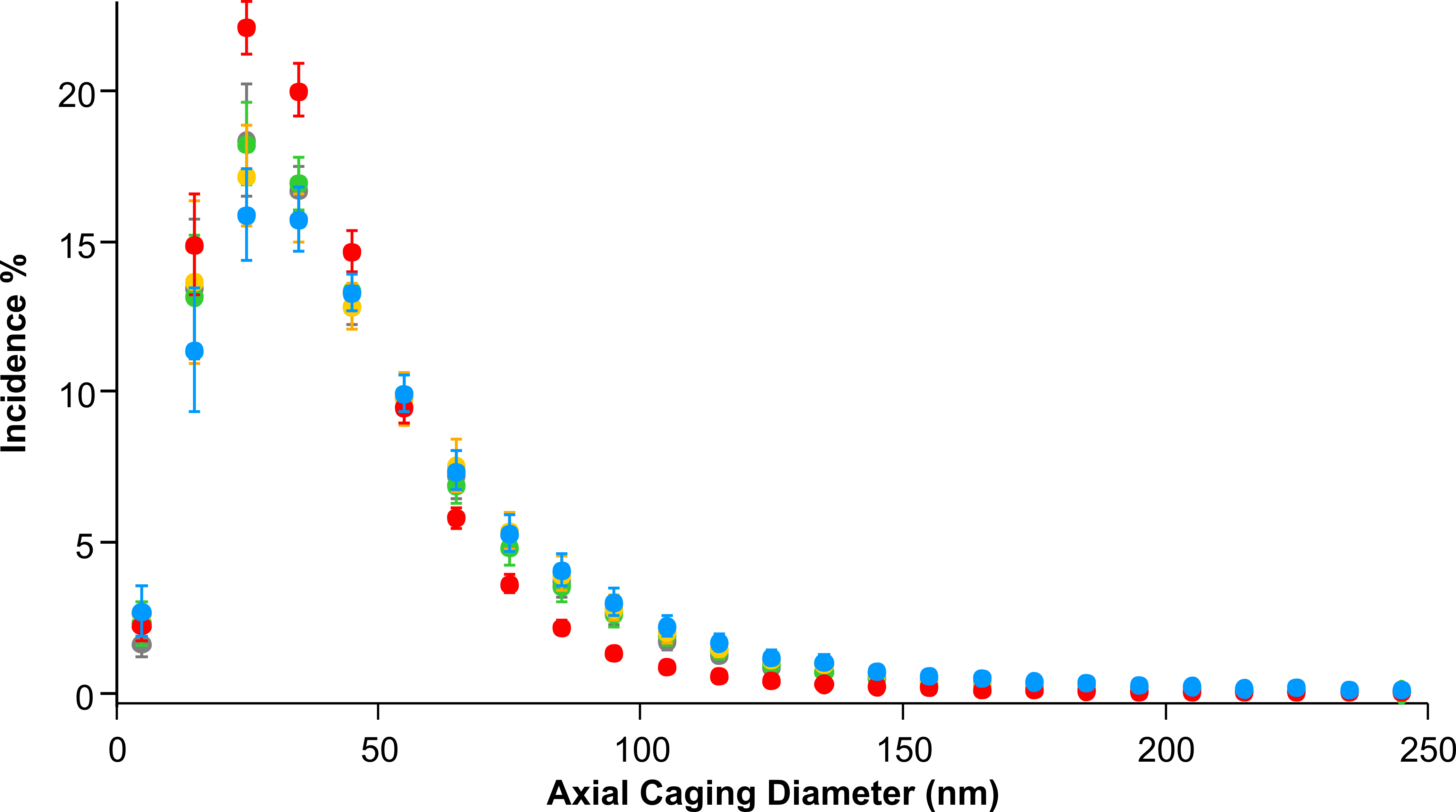

Supplement: Figure S3 — Axial CD frequency distribution with SEM. blue circles, 100 nM; green circles, 300 nM; red circles, 500 nM; orange circles, 700 nM; gray circles, >800 nM [Ca2+]i). (TIF) [file pone.0036416.s003.tif]

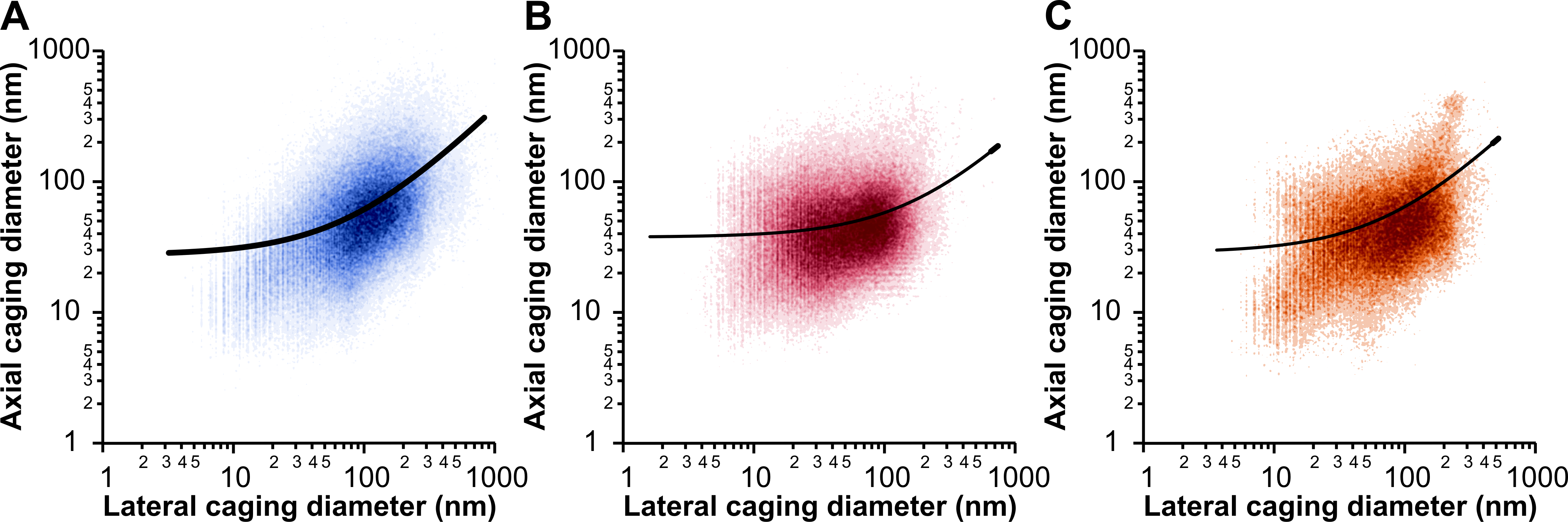

Supplement: Figure S4 — Lateral vs. axial CD correlation plot. (A, B, C) To analyze the correlation of lateral and axial mobility, the CD of each LDCV which was used for the analysis of the axial movements was plotted versus its lateral CD on a double-logarithmic scale: (A) 100 nM, (B) 500 nM and (C) 700 nM. A large number of points overlap. To visualize this, the density of the CDs is color encoded. The more points are present on one spot the darker becomes this spot. The plots were fitted by a linear curve (f(x) = a+bx) and the slope b is shown on each curve. Note that this slope was specifically reduced at 500 nM free Ca2+. This indicates that the mobility of LDCVs from and to the PM is decreased at lower [Ca2+]i than their mobility along the PM (blue circles, 100 nM; red circles, 500 nM; orange circles, 700 nM [Ca2+]i). (TIF) [file pone.0036416.s004.tif]

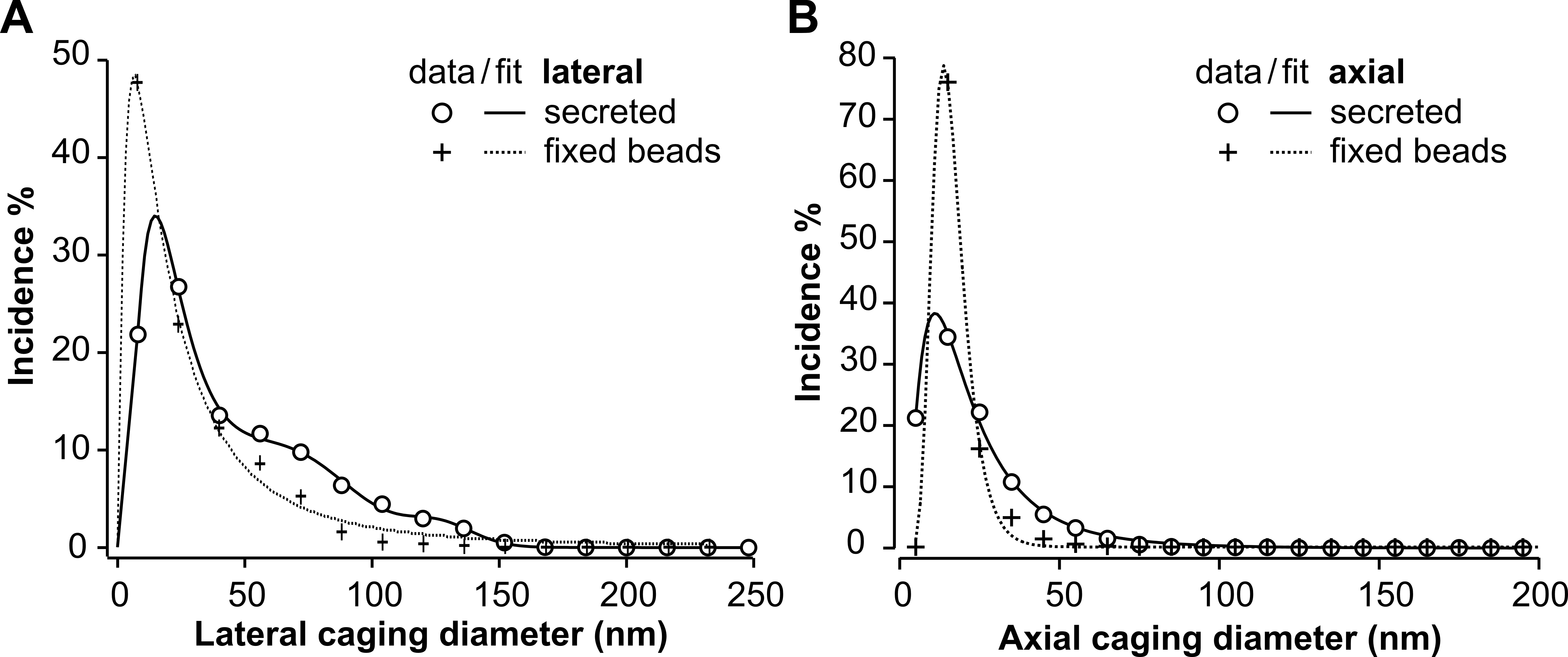

Supplement: Figure S5 — Comparison between lateral or axial CD histogram of secreted vesicles and fixed fluorescent beads. (A) Lateral CD histogram of secreted vesicles (solid line) and fixed beads (dashed line). (B) Axial CD histogram of secreted vesicles (solid line) and fixed beads (dashed line). Note that in contrast to the lateral histogram the shape of the axial CD histogram of secreted vesicles is similar to the shape of the axial histogram of fixed beads. (TIF) [file pone.0036416.s005.tif]
