# Supplementary material for: Docking of LDCVs Is Modulated by Lower Intracellular [Ca2+] than Priming
Source: PLoS One. 2012 May 10;7(5):e36416. doi: 10.1371/journal.pone.0036416 (PMC3349663; doi:10.1371/journal.pone.0036416)
Supplement: Table S1 — Statistical analysis of the fluorescence intensity vs. residence time correlation plot. RT stands for residence time; NMFI stands for normalized median fluorescence intensity. (DOC) [file pone.0036416.s006.doc]

|  | **Overall % LDCV** | | **% LDCVs within a Residence time range** | |
| --- | --- | --- | --- | --- |
|  | Average | SEM | Average | SEM |
| RT<1s AND NMFI<0.2 | 26.1 | 4.2 | 81.0 | 8.0 |
| RT<1s AND 0.2≤NMFI<0.5 | 5.0 | 2.0 | 19.0 | 8.0 |
| RT<1s AND 0.5≤NMFI | 0.0 | 0.0 | 0.0 | 0.0 |
| Subtotal | 31.1 |  | 100 |  |
| 1s≤RT<5s AND NMFI<0.2 | 19.7 | 3.8 | 68.9 | 9.8 |
| 1s≤RT<5s AND 0.2≤NMFI<0.5 | 7.5 | 2.3 | 31.1 | 9.8 |
| 1s≤RT<5s AND 0.5≤NMFI<1 | 0.0 | 0.0 | 0.0 | 0.0 |
| Subtotal | 27.2 |  | 100 |  |
| 5s≤RT AND NMFI<0.2 | 9.9 | 3.0 | 26.6 | 8.6 |
| 5s≤RT AND 0.2≤NMFI<0.5 | 23.1 | 4.4 | 52.1 | 8.4 |
| 5s≤RT AND 0.5≤NMFI | 8.7 | 1.9 | 21.3 | 5.6 |
| Subtotal | 41.8 |  | 100 |  |
| Sum | 100 |  |  |  |
