# Supplementary material for: Docking of LDCVs Is Modulated by Lower Intracellular [Ca2+] than Priming
Source: PLoS One. 2012 May 10;7(5):e36416. doi: 10.1371/journal.pone.0036416 (PMC3349663; doi:10.1371/journal.pone.0036416)
Supplement: Table S2 — Statistical analysis over lateral CD distribution. P value obtained through two way ANOVA on Rank using Holm-Sidak post-test.100 nM: N = 23, n = 719; 300 nM: N = 13, n = 403; 500 nM: N = 26, n = 695; 700 nM N = 14, n = 309; >800 nM: N = 16, n = 452. (DOC) [file pone.0036416.s007.doc]

| **[Ca^2+^]_i_ (nM)** | **Value of the bin's center of the lateral CD histogram (nm)** | | | | | | | | | | | | | | | | | |
| --- | --- | --- | --- | --- | --- | --- | --- | --- | --- | --- | --- | --- | --- | --- | --- | --- | --- | --- |
|  | **8** | **24** | **40** | **56** | **72** | **88** | **104** | **120** | **136** | **152** | **168** | **184** | **200** | **216** | **232** | **248** | **264** | **280** |
| 100 vs 300 | ns | **<0.001** | **<0.001** | ns | ns | ns | ns | ns | ns | ns | ns | ns | ns | ns | ns | ns | ns | ns |
| 100 vs 500 | ns | **<0.001** | **<0.001** | 0.038 | ns | ns | ns | ns | ns | ns | ns | ns | ns | ns | ns | ns | ns | **ns** |
| 100 vs 700 | **0.001** | **<0.001** | **<0.001** | ns | **<0.001** | ns | 0.007 | ns | ns | ns | ns | ns | ns | ns | ns | ns | ns | ns |
| 100 vs >800 | ns | **<0.001** | **<0.001** | ns | **<0.001** | 0.002 | ns | ns | ns | ns | ns | ns | ns | ns | ns | ns | ns | ns |
| 300 vs 500 | ns | ns | ns | ns | ns | ns | ns | ns | ns | ns | ns | ns | ns | ns | ns | ns | ns | ns |
| 300 vs 700 | **<0.001** | **<0.001** | ns | ns | ns | ns | ns | ns | ns | ns | ns | ns | ns | ns | ns | ns | ns | ns |
| 300 vs >800 | 0.035 | ns | ns | ns | ns | ns | ns | ns | ns | ns | ns | ns | ns | ns | ns | ns | ns | ns |
| 500 vs 700 | 0.021 | ns | ns | ns | 0.006 | ns | ns | ns | ns | ns | ns | ns | ns | ns | ns | ns | ns | ns |
| 500 vs >800 | ns | ns | ns | ns | 0.015 | 0.011 | ns | ns | ns | ns | ns | ns | ns | ns | ns | ns | ns | ns |
| 700 vs >800 | ns | ns | ns | ns | ns | ns | ns | ns | ns | ns | ns | ns | ns | ns | ns | ns | ns | ns |
