# Supplementary material for: Docking of LDCVs Is Modulated by Lower Intracellular [Ca2+] than Priming
Source: PLoS One. 2012 May 10;7(5):e36416. doi: 10.1371/journal.pone.0036416 (PMC3349663; doi:10.1371/journal.pone.0036416)
Supplement: Table S3 — Statistical analysis over axial CD distribution. P value obtained through two way ANOVA on Rank using Holm-Sidak post-test. 100 nM: N = 23, n = 719; 300 nM: N = 13, n = 403; 500 nM: N = 26, n = 695; 700 nM N = 14, n = 309; >800 nM: N = 16, n = 452. (DOC) [file pone.0036416.s008.doc]

| **[Ca^2+^]_i_ (nM)** | **Value of the bin's center of the axial CD histogram (nm)** | | | | | | | | | | | | | | | | | | | | | |
| --- | --- | --- | --- | --- | --- | --- | --- | --- | --- | --- | --- | --- | --- | --- | --- | --- | --- | --- | --- | --- | --- | --- |
|  | **5-15** | **25** | **35** | **45** | **55** | **65** | **75** | **85** | **95** | **105** | **115** | **125** | **135** | **145** | **155** | **165** | **175** | **185-195** | **205** | **215** | **225** | **235** |
| 100 vs 300 | ns | ns | 0.015 | ns | ns | ns | ns | ns | ns | ns | ns | ns | ns | ns | ns | ns | ns | ns | ns | ns | ns | ns |
| 100 vs 500 | ns | **<0.001** | **<0.001** | **<0.001** | ns | ns | ns | ns | 0.029 | ns | ns | ns | ns | ns | ns | ns | ns | ns | ns | ns | ns | ns |
| 100 vs 700 | ns | 0.017 | ns | ns | ns | ns | ns | ns | ns | ns | ns | ns | ns | ns | ns | ns | ns | ns | ns | ns | ns | ns |
| 100 vs >800 | ns | 0.045 | 0.005 | ns | ns | ns | ns | ns | ns | ns | ns | ns | ns | ns | ns | ns | ns | ns | ns | ns | ns | ns |
| 300 vs 500 | ns | ns | **<0.001** | **<0.001** | ns | ns | ns | ns | ns | ns | ns | ns | ns | ns | ns | ns | ns | ns | ns | ns | ns | ns |
| 300 vs 700 | ns | ns | ns | ns | ns | ns | ns | ns | ns | ns | ns | ns | ns | ns | ns | ns | ns | ns | ns | ns | ns | ns |
| 300 vs >800 | ns | ns | ns | ns | ns | ns | ns | ns | ns | ns | ns | ns | ns | ns | ns | ns | ns | ns | ns | ns | ns | ns |
| 500 vs 700 | ns | **ns** | **<0.001** | **<0.001** | ns | ns | ns | ns | ns | ns | ns | ns | ns | ns | ns | ns | ns | ns | ns | ns | ns | ns |
| 500 vs >800 | ns | ns | **<0.001** | ns | ns | ns | ns | ns | ns | ns | ns | ns | ns | ns | ns | ns | ns | ns | ns | ns | ns | ns |
| 700 vs >800 | ns | ns | ns | ns | ns | ns | ns | ns | ns | ns | ns | ns | ns | ns | ns | ns | ns | ns | ns | ns | ns | ns |
